# A Star-Shaped Copolymer with Tetra-Hydroxy-Phenylporphyrin Core and Four PNIPAM-*b*-PMAGA Arms for Targeted Photodynamic Therapy

**DOI:** 10.3390/polym15030509

**Published:** 2023-01-18

**Authors:** Changling Liu, Yirong Wang, Siyu Wang, Pengcheng Xu, Renning Liu, Dandan Han, Yen Wei

**Affiliations:** 1 School of Materials Science and Engineering, Jilin Institute of Chemical Technology, Jilin City 132022, China; 2 College of Biology and Food Engineering, Jilin Institute of Chemical Technology, Jilin City 132022, China; 3 Department of Chemistry, The Tsinghua Center for Frontier Polymer Research, Tsinghua University, Beijing 100084, China

**Keywords:** porphyrin, THPP-(PNIPAM-*b*-PMAGA)_4_, thermosensitivity, photodynamic therapy, targeted drug delivery

## Abstract

The novel thermosensitive star-shaped tetra-hydroxy-phenylporphyrin-cored (THPP) double hydrophilic poly(N-isopropylacrylamide)-b-poly(methylacrylamide glucose) block copolymers (THPP-(PNIPAM-*b*-PMAGA)_4_) were synthesized via the reversible addition-fragmentation chain transfer (RAFT) polymerization. Notably, the low critical solution temperatures (LCSTs) of THPP-(PNIPAM-*b*-PMAGA)_4_ were above normal body temperature (37 °C) which depended on the hydrophilic PMAGA contents of copolymers. When the temperature was higher than the LCST of the copolymer, the copolymer could be neutralized into micelles in aqueous and could be coated with antitumor drugs and released around tumor cells. The MTT study indicated that THPP-(PNIPAM-*b*-PMAGA)_4_ had a low toxicity to L929 and HeLa cells in the absence of light. However, THPP-(PNIPAM-*b*-PMAGA)_4_ showed a high toxicity with HeLa cells under light irradiation which could be used as a potential photosensitizer for photodynamic therapy (PDT). In addition, THPP-(PNIPAM-*b*-PMAGA)_4_ showed specific a recognition function with Concanavalin A (Con A) to achieve active targeted drug delivery. This work provides a new approach for the development of tumor targeting and chemotherapy/PDT.

## 1. Introduction

Porphyrins and their derivatives have attracted extensive attention in the field of designing and synthesizing novel functional materials. However, most of porphyrin’s derivatives were hydrophobic, non-selective, and self-quenching [1,2,3,4,5,6,7,8,9,10,11,12,13,14]. Up to the present, water-soluble porphyrins without charge were not easy to obtain, and almost all had ionic structure [15]. In recent years, many studies have addressed the above shortcomings by modifying porphyrins in liposomes, nanoparticles, and polymers [16,17,18,19,20,21,22,23,24]. Frechet et al. synthesized a functional porphyrin-cored polymeric shell using Ring Opening Polymerization (ROP) of 3-caprolactone (CL) or L-lactic (L-LA) [25]. Holder and Cornelissen et al. reported a series of star polymers with porphyrin cores by Atom Transfer Radical Polymerization (ATRP). Among these, the modified porphyrins by hydrophilic polymers could effectively improve the solubility of porphyrin [26,27,28]. The hydrophilic compounds could be coupled with porphyrin to a PDT-like hyaluronic acid, chondroitin sulfate, pluronic F127, and polyethylenimine [29,30]. Nicosia et al. combined the bactericidal and photothermal properties of AgNPs with the characteristics of PEGylated porphyrins [31]. Among porphyrin polymers, Poly (*N*-isopropylacrylamide) (PNIPAM) based on polymer-porphyrin has attracted a great deal of attention due to the temperature responsiveness of PNIPAM. Xu et al. [32] prepared amphiphilic PNIPAM-b-PTPPC6MA block copolymers containing porphyrin. Our group has also successfully synthesized a series of porphyrin-based PNIPAM for photodynamic therapy [33,34]. These porphyrin-modified thermosensitive polymers can self-assemble into micelles which could be used as PDT agents and drug carriers [35,36,37]. However, none of them had the ability to recognize the active targeting of malignant tissue. Glycosylation can offer the possibility of specific interactions with lectin-type receptors that are overexpressed in specific malignant tissues. D-glucosamine (GA) has good hydrophilicity, biocompatibility, and biodegradation and also has an excellent cell penetrating ability, immunomodulatory properties, and a strong tumor cell killing ability [38]. Until now, Dong et al. synthesized a serial of hydrophilic chains composed of some glycopolymers which provided a specific sugar-protein recognition processes [39]. Dai et al. prepared a biomimetic poly (3-caprolactone)-bglycopolymer block copolymers with porphyrin, but also provided a very specific recognition with Concanavalin A (Con A) [40].

**Scheme 1:**
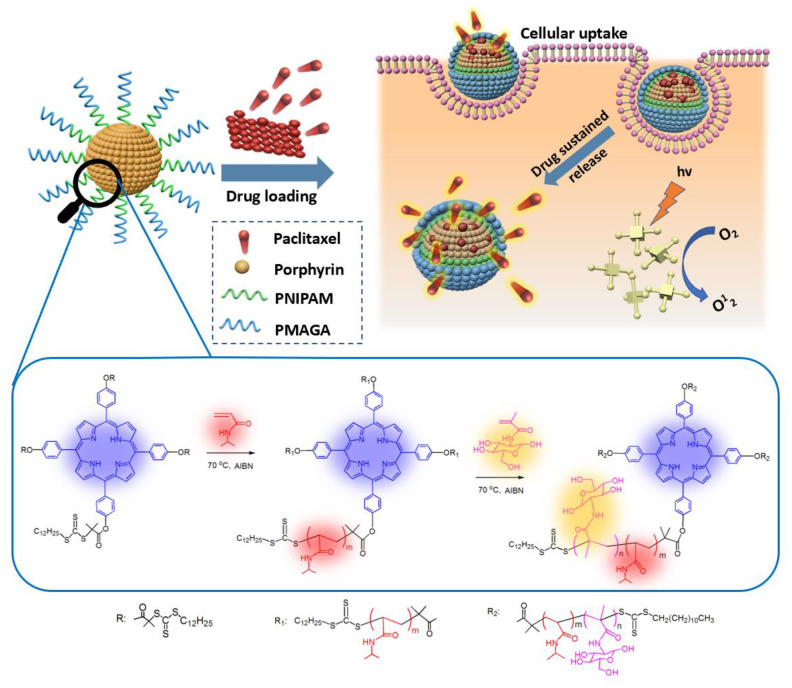
Schematic diagram of PDT mechanism and synthesis of THPP-(PNIPAM-b-PMAGA)_4_-1(*M*_n_ = 18,700)/paclitaxel.

We have previously synthesized several polymers with PNIPAM and porphyrin that self-assemble into micelles in organic solvents. Here, our research group designed and synthesized star-shaped double hydrophilic THPP-(PNIPAM-*b*-PMAGA)_4_ containing a free tetra-hydroxy-phenylporphyrin core and four poly (*N*-isopropylacrylamide)-b- poly (methacrylamide Glucose) (PNIPAM-*b*-PMAGA) arms via RAFT polymerization. Moreover, the reaction process is shown Scheme 1. This compound did not require an organic solvent and can form micelles directly in water.

When the temperature was higher than LCST, the PNIPAM segment of THPP-(PNIPAM-*b*-PMAGA)_4_ changed from hydrophilic to hydrophobic, while the PMAGA segment remained hydrophilic and soluble, resulting in the formation of micelles in the aqueous solution of THPP-(PNIPAM-*b*-PMAGA)_4_. By adjusting the LCST of the copolymer, the hydrophilic PNIPAM chain segment became hydrophobic and released drugs in the tumor tissue. Consequently, it provided not only a specific sugar recognition and temperature-targeting block copolymer, but also a potential porphyrin-cored star-shaped PNIPAM-*b*-PMAGA copolymers for combination chemotherapy/PDT strategies.

## 2. Materials and Methods

### 2.1. Materials

N-isopropylacrylamide (NIPAM) was purchased from Aldrich (Sigma-Aldrich Trading Co, Ltd, Shanghai, China) and recrystallized to remove the polymer inhibitor. The monomer methacrylamide glucose was synthesized according to the literature [41]. *S*-1-Dodecyl-*S*′-(α, α′-dimethyl-*α″*-acetic acid) trithiocarbonate (DMP) as chain transfer agent of RAFT was synthesized according to the literature [42,43]. All other chemicals were purchased from Aldrich with analytical grade and used directly.

### 2.2. Characterization

The structures of the products were characterized by Fourier Transform infrared (FT-IR) spectra recorded on Shimadzu-8400S (Shimadzu Corporation, Tokyo, Japan)infrared spectrometer, nuclear magnetism resonance spectroscopy (NMR) which recorded on Bruker Avance 400 MHz spectrometer(Bruker, Schlieren, Switzerland), and gel permeation chromatography (GPC) which was measured by Waters e2695(Waters, Milford, MA, USA). The thermal response of the copolymer was measured by shimadu-1240 UV–visible spectrophotometer with an external temperature controller. The dynamic light scattering (DLS) of 2 mg·mL^−1^ THPP-(PNIPAM-*b*-PMAGA)_4_ was measured on a Malvern Nano S particle analyzer (Malvern Panalytical Ltd, Malvern, UK) in the temperature range of 25 °C to 45 °C. The CMC of polymer was determined by fluorescence spectrophotometer [44].

### 2.3. The Synthesis of THPP-(PNIPAM-b-PMAGA)_4_ Copolymers

THPP-(PNIPAM-*b*-PMAGA)_4_ containing porphyrin core, PNIPAM, and PMAGA were synthesized by RAFT. The tetra-hydroxy-phenylporphyrin was synthesized according to the literature [45]. Firstly, THPP-(DMP)_4_ was synthesized by esterification of THPP core with chain transfer agent catalyzed by DCC and DMAP. Subsequently, the THPP-(PNIPAM)_4_ was synthesized by RAFT polymerization.

**Figure 1:**
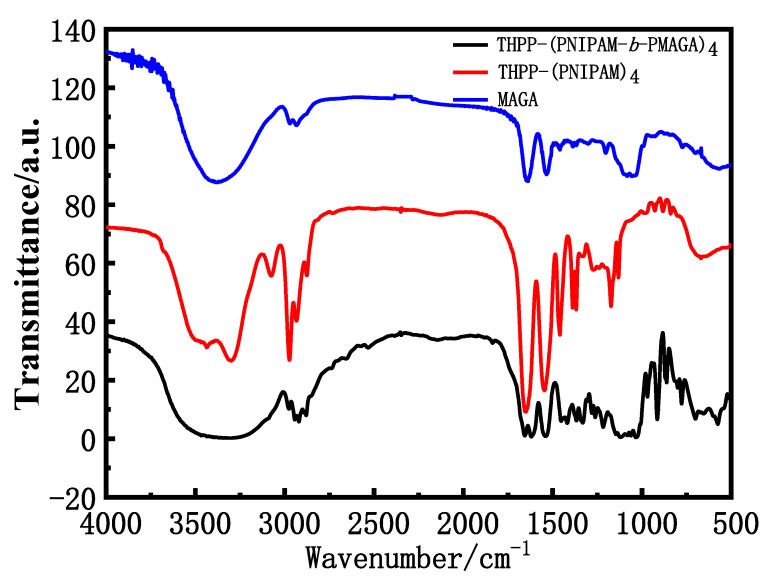
FTIR spectra of MAGA, THPP-(PNIPAM)_4_ and THPP-(PNIPAM-*b*-P MA GA)_4_.

**Figure 2:**
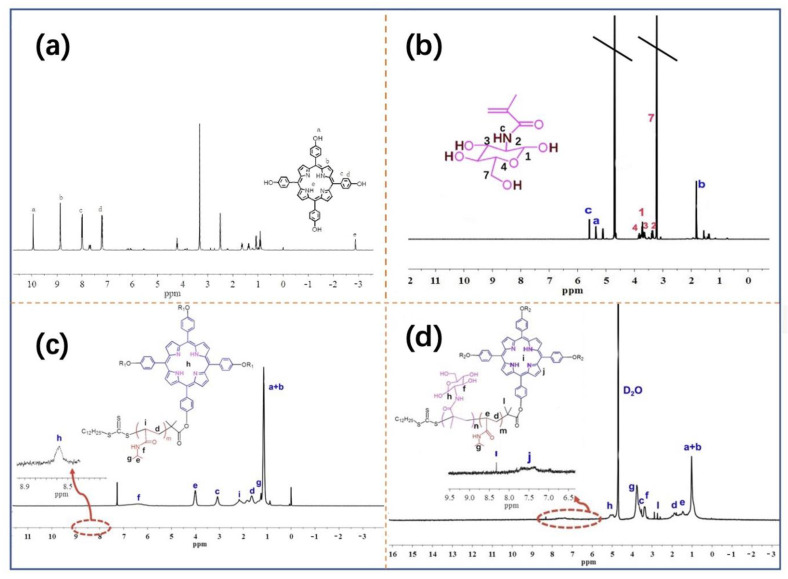
(**a**) ^1^HNMR spectrum of THPP in DMSO; (**b**) ^1^HNMR spectra of MAGA in D_2_O; (**c**) ^1^HNMR spectra of THPP-(PNIPAM)_4_ in CDCl_3_; (**d**) ^1^HNMR spectra of THPP-(PNIPAM-*b*-PMAGA)_4_ in CDCl_3_.

In short, a certain amount of THPP-(DMP)_4_ was added into DMF (2 mL) solution of 226 mg NIPAM and stirred to deoxygenate. Then, 3.3 mg AIBN was added into oil bath at 70 °C for 24 h. The product was concentrated and precipitated in ice ether and dried under vacuum. *M*_n,GPC_ = 10,500, *M*_w_/*M*_n_ = 1.15. FTIR: 1640 (*ν*_C = O_), 1540 (*δ*_C-N-H_) [Figure 1]. ^1^HNMR (400 MHz, CDCl_3_), *δ* ppm: 8.8–8.2 (porphyrin-H), 6.26 (t,-NH), 4.0 (m,-NCH) [Figure 2c].

D- (+) -glucosamine hydrochloride (5.00 g, 28 mmol) and potassium carbonate (4.2 g, 30 mmol) were added to 125 mL of methanol and cooled to −15 °C. The methylacrylyl chloride (3.12 g, 30 mmol) drop by drop to the reactor and the reaction was performed at −15 °C for 30 min. The reaction was continued at 25 °C for 3 h. After the reaction, the filtrate was filtered and spun to the suspension. The suspension was purified by silica gel column chromatography (the V_CH2Cl2_:V_CH3OH_ = 4:1) and the white solid was obtained. FT-IR (KBr, cm^−1^): 1664 cm^−1^(*ν*_-CONH_), 1542 cm^−1^(*ν*_-N-H_), 2937 cm^−1^, 2862 cm^−1^ (*ν*_-C-H_) [Figure 1]. ^1^H NMR (400 MHz, D_2_O) δ 5.6 (s, 1H, -N*H*), 5.4 (s, 2H, =C*H_2_*), 3.2 (s, 4H, -O*H*), 3.88–3.43 (s, 5H, -C*H-*OH), 1.76 (s, 3H, -C*H_3_*) [Figure 2a].

THPP-(PNIPAM-*b*-PMAGA)_4_ were prepared via RAFT using THPP-(PNIPAM)_4_ as chain transfer agent. THPP-(PNIPAM)_4_ of 1.05 g, MAGA of 0.49 g, and AIBN of 3.3 mg were dissolved in 5 mL of DMF and the reaction was performed at 65 °C for 12 h after stirring and deoxygenation. Polymer products were dialysed in water using dialysis bags (MWCO, 12,000) at 6-h intervals for two days and freeze-dried for storage. *M*_n,GPC_ = 30,200, *M*_w_/*M*_n_ = 1.3. FT-IR (KBr, cm^−1^): 1643 cm^−1^(*ν*_-CONH_), 1525 cm^−1^(*ν*_-N-H_), 3392 cm^−1^(*ν*_-O-H_) [Figure 1]. ^1^H NMR (400 MHz, D_2_O): δ 7.3 (s, -N*H*-C (CH_3_)_2_), 8.35 (s, -N*H*-MAGA), 4.0 (m, -NC*H*), 3.4 (s, -SC*H_2_*), 3.0–5.0 ppm (m, MAGA-C*H*), 1.03 (m, -C*H_2_*C*H_3_*) [Figure 2c].

### 2.4. The Preparation of THPP-(PNIPAM-b-PMAGA)_4_ Micelles

Using pyrene as a fluorescent probe, a certain amount of pyrene was added into acetone solvent, and 0.1 mL pyrene-acetone solution was added into 10 mL volumetric bottles respectively [46]. The acetone solvent was volatilized at room temperature. Then 10 mL of polymer aqueous solution with different concentrations were added to above volumetric bottles, and the final concentration of pyrene reached 2 × 10^−6^ mol/L. The emission spectrum of the copolymer in the range of 340~600 nm was measured at an excitation wavelength of 335 nm. The CMC was taken as the cross-point when extrapolating the intensity ratio *I*383/*I*372 at low-and high-concentration regions.

### 2.5. Singlet Oxygen Generation

In this study, the ^1^O_2_ quantum yield was determined by mixing 1,3-diphenylisobenzofuran (DPBF) with THPP-(PNIPAM-*b*-PMAGA)_4_, the DPBF decay at 410 nm was measured by UV–visible spectrophotometer and monitored every 1 min.

The *Φ*_Δ_ was calculated according to Equation (1):

(1) $$\Phi_{\Delta }=\Phi_{\Delta }^{Std}\frac{RI_{abs}^{Std}}{R^{Std}I_{abs}}$$

Among them, $\Phi_{\Delta }^{Std}$ is 0.62 of the ^1^O_2_ quantum yield for TPP. The DPBF photobleaching rates of copolymers and TPP are *R* and *R^Std^*, respectively, while $I_{abs}$ and $I_{abs}^{Std}$ are their photoabsorption rates.

### 2.6. Cell Biology Experiment of THPP-(PNIPAM-b-PMAGA)_4_ Micelles

The toxicity of THPP-(PNIPAM-*b*-PMAGA)_4_ micelles to different cells was determined by methylthiazoltetrazole (MTT) method without light irradiation. Fetal bovine serum (FBS, 10% (v)), penicillin (100 units mL^−1^), and streptomycin (100 μg·mL^−1^) were added to all cells by adding amino acid and glucose medium to the 96-well flat plate.

At 37 °C, the cells were incubated for 48 h, washed with PBS 3 times, incubated for 6 h, added 20 μL MTT solution containing micelle solution of different concentrations for 4 h, and then returned to the incubator. Carefully, the MTT solution was taken out, 200 μL DMSO was added and gently stirred for 10 min and the formazan product was extracted. Absorbance at 492 nm was read with an enzyme-linked immunosorbent assay (ELISA) microplate reader (Bio-Rad). According to Formula (2), cell viability (%) can be calculated.

Cell viability (%) = (A_sample_/A_control_) × 100% (2)

At the same time, the phototoxicity of THPP-(PNIPAM-*b*-PMAGA)_4_ micelles on cells were measured by red light irradiation at 670 nm for 5 min according to the above method.

HeLa cells were cultured in 96-well plates containing 2 mL medium for 24 h with a cell density of 1 × 10^6^ cells per well. The cells were then treated with samples for a certain period of time and washed with phosphate buffered saline (PBS). Finally, the expected precipitation was analyzed by BD FACS Calibur flow cytometry. The data were analyzed by FlowJo software.

### 2.7. Lectin Recognition

The Con A lectin recognition behavior of the sugar-containing copolymer solution was determined by UV–VIS method at 360 nm. In addition, the concentration of Con A was set as 0.5 mg·mL^−1^, and different concentrations of aggregate solution were added to the Con A solution.

## 3. Results and Discussion

### 3.1. Analysis of Copolymers Architecture

The architecture of the THPP-(PNIPAM-b-PMAGA)4 was authenticated by FTIR spectra and ^1^HNMR. Figure S1 shows O-H absorbance and N-H tensile vibration in THPP at 3430 cm^−1^ and 3350 cm^−1^, respectively. From Figure 2a, the chemical shift of 9.96, 8.65, and −2.86 ppm were the characteristic signals of THPP’s hydroxyl group, benzene ring, and amino group (Figure S1 and Figure 2a). The absorbance at 1760 cm^−1^ was ascribed to the ester group of THPP-(DMP)_4_ (Figure S2). In Figure S3, ^1^HNMR spectra of THPP-(DMP)_4_ showed that (δ~3.4) and (δ~0.98) were assigned to the proton of -CH_2_S and -CH_3_. The FTIR and ^1^HNMR spectra of Methylacrylamide glucose (MAGA) are shown in Figure 1 and Figure 2a. The peak at 2937 cm^−1^ was the methyl antisymmetric stretching vibration peak, 2920 cm^−1^ and 2955 cm^−1^ were the methylene C-H stretching vibration peak, the peaks at 1664 cm^−1^ and 1542 cm^−1^ were vested in the stretching vibration peak of the carbon oxygen double bond on MAGA, which were the characteristic absorption peaks of amide I band and the characteristic absorption peaks of N-H bending vibration coupling with C-N stretching vibration in CONH, namely the amide II band (Figure 1). In Figure 2b, two peaks at about 1.6 ppm, 5.5 ppm, and 5.7 ppm were attributed to protons of the methyl group attached to the double bond, and the MAGA double bond and N-H, respectively. The target product MAGA was successfully characterized by FTIR and ^1^HNMR.

The FTIR spectrum of THPP-(PNIPAM-b-PMAGA)_4_ was shown in Figure 1. The absorption peaks of the amide I and II bands in PNIPAM were at 1643 cm^−1^ and 1552 cm^−1^, respectively. After copolymerization with MAGA, the presence of a large number of hydroxyl groups in the glucose molecule resulted in a broad and strong hydroxyl absorption peak near 3320 cm^−1^. From the ^1^HNMR of Figure 2c, the acylamino groups and methenyl adjacent to acylamino of PNIPAM were located at 6.0–7.0 ppm and 4.0 ppm. In Figure 2d, the new signal at 3.0–5.0 ppm was corresponding to protons in PMGA. Figure S4 shows the GPC curve of the polymer. The polymerization data of THPP-(PNIPAM)_4_ and THPP-(PNIPAM-*b*-PMAGA)_4_ is shown in Table 1. All the above test results confirmed that THPP-(PNIPAM-*b*-PMAGA)_4_ copolymers were synthesized successfully [47,48].

### 3.2. Photophysical Properties of THPP-(PNIPAM-b-PMAGA)_4_

**Figure 3:**
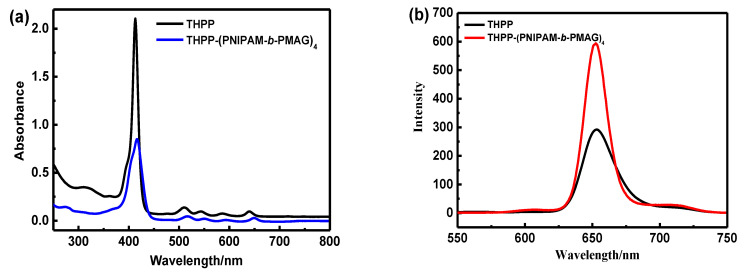
(**a**) UV–Vis spectra of free THPP and THPP-(PNIPAM-*b*-PMAGA)_4_ in CH_3_OH solution; (**b**) emission spectra of free THPP and THPP-(PNIPAM-*b*-PMAGA)_4_ in CH_3_OH solution (0.125 mg mL^−1^).

Figure 3a showed the ultraviolet absorption spectra of THPP and THPP-(PNIPAM-*b*-PMAGA)_4_ in methanol solution. THPP-(PNIPAM-*b*-PMAGA)_4_ and THPP both had B-band absorption peaks at about 420 nm and four weak Q-band absorption peaks at 500–700 nm. However, THPP-(PNIPAM-*b*-PMAGA)_4_ had a red shift which was caused by the narrowing of the band gap due to the long polymer chain and the easy excitation of electrons.

Fluorescence emission spectra of THPP and THPP-(PNIPAM-*b*-PMAGA)_4_ were illustrated in Figure 3b. At the excitation wavelength of 380 nm, the maximum emission peak was around 650 nm, but the charge-transfer effect of THPP-(PNIPAM-*b*-PMAGA)_4_ was reduced due to the presence of long polymer chains, and therefore, the emission band was enhanced and potentially split [49,50].

### 3.3. Thermosensitive Behavior of THPP-(PNIPAM-b-PMAGA)_4_

**Figure 4:**
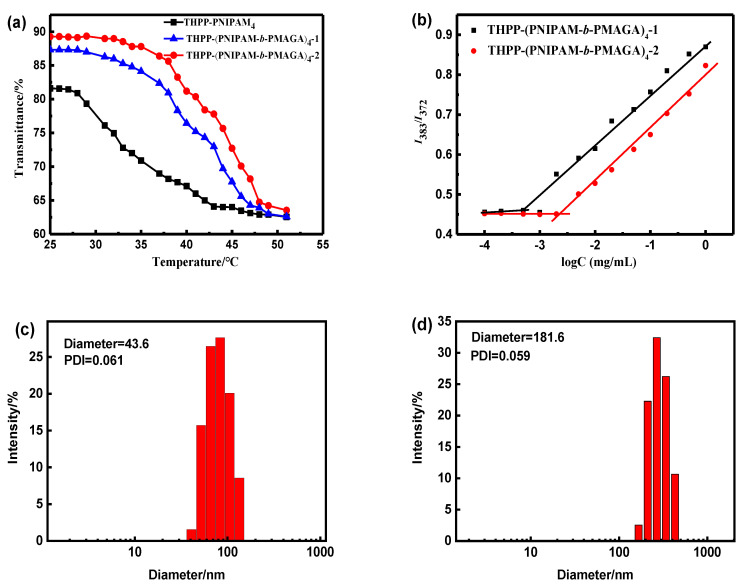
(**a**,**b**) Temperature dependence of optical transmittance at wavelength of 500 nm obtained for aqueous solutions and Plot of the I_383_/I_372_ ratio against log C of THPP-PNIPAM_4_ and THPP-(PNIPAM-b-PMAGA)_4_^−1^; (**c**) Average size of THPP-(PNIPAM-b-PMAGA)_4_ micelles at 35 °C; (**d**) Average size of THPP-(PNIPAM-b-PMAGA)_4_ micelles 45 °C (the concentration of copolymer was 1 mg·mL^−1^).

Figure 4a shows the LCST curve of THPP-(PNIPAM-*b*-PMAGA)_4_ copolymers in aqueous solution. The LCST of copolymer THPP-(PNIPAM-*b*-PMAGA)_4_ with the introduction of hydrophilic MAGA segment was higher than that of THPP-PNIPAM_4_ [51,52] (Table 2). The LCST increased with the increase in hydrophilic MAGA segments. Figure 3c,d shows the dynamic radius and distribution of THPP-(PNIPAM-*b*-PMAGA)_4_ micelles at 35 °C and 45 °C.

It was shown that the particle size of a THPP-(PNIPAM-b-PMAGA)_4_ micelle was 43.6 nm when the temperature was 35 °C, but the particle size increased to 181.6 nm when the temperature was increased to 45 °C in Figure 4. The results revealed that when the temperature was higher than the LCST of the copolymer, the PNIPAM segment changed from hydrophilic to hydrophobic and the completed collapse of hydrogen bonds led to an increase in the particle size of the formed micelles.

### 3.4. CMC of THPP-(PNIPAM-b-PMAGA)_4_

The CMC of THPP-(PNIPAM-*b*-PMAGA)_4_ are shown in Figure 4b. With the increasing of THPP-(PNIPAM-*b*-PMAGA)_4_ concentration, the ratio of *I*383/*I*372 remained unchanged, but it increased sharply when the copolymer concentration gradually increased to a certain value. It indicated that hydrophobic pyrene accumulates to the micelle core, which was a sign of micelle formation. The CMC value of THPP-(PNIPAM-*b*-PMAGA)_4_ copolymer increased from 0.0005 mg·mL^−1^ to 0.0025 mg·mL^−1^ with the increasing of the hydrophilic PMAGA segment. It was confirmed that the polymer micelle might be formed with the increase in the length of hydrophobic block.

### 3.5. Singlet Oxygen Quantum Yields of THPP-(PNIPAM-b-PMAGA)_4_

**Figure 5:**
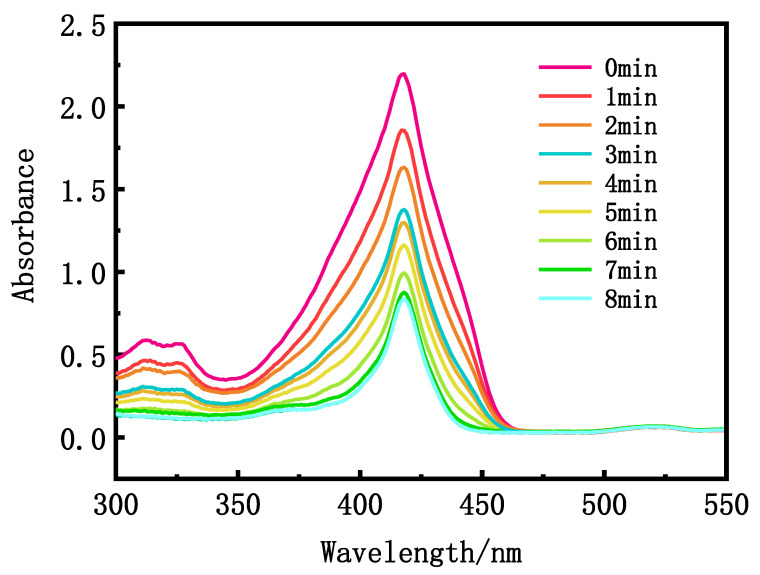
UV–vis spectra for the determination of singlet oxygen generation quantum yield of THPP-(PNIPAM-*b*-PMAGA)_4_ in DMF use DPBF as quencher (λ = 410 nm).

The absorbance intensity of DPBF and the THPP-(PNIPAM-*b*-PMAGA)_4_ mixture in DMF determined by a UV–UV spectrophotometer is shown in Figure 5. The *Φ*_Δ_ of THPP-(PNIPAM-*b*-PMAGA)_4_ was estimated from Equation (1). The *Φ*_Δ_ of THPP-(PNIPAM-*b*-PMAGA)_4_ with different molecular weights in DMF were 0.41 and 0.37, respectively. In conclusion, THPP-(PNIPAM-*b*-PMAGA_)4_ might be a promising reagent for PDT.

### 3.6. Dark Cytotoxicity and Phototoxicity of THPP-(PNIPAM-b-PMAGA)_4_

**Figure 6:**
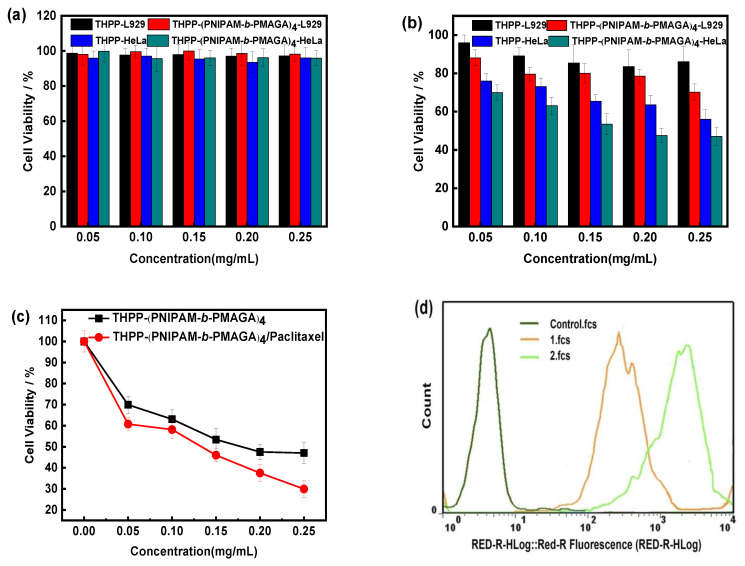
Viability of L929 cells and HeLa cells determined by the MTT assay after treatment of THPP and THPP-(PNIPAM-*b*-PMAGA)_4_ micelles: (**a**) dark toxicity; (**b**) phototoxicity; (**c**) Viability of HeLa cells determined by the MTT assay after treatment of THPP-(PNIPAM-*b*-PMAGA)_4_ and THPP-(PNIPAM-*b*-PMAGA)_4_/paclitaxel micelles; (**d**) Flow cytometric demonstration of HeLa cells after incubation with control (dark green line), THPP for 4 h (brown line), THPP-(PNIPAM-*b*-PMAGA)_4_ for 12 h (green line).

Figure 6a shows the dark-cytotoxicity of THPP, THPP-(PNIPAM-*b*-PMAGA)_4_ to normal cell L929 and human cervical cancer cell HeLa measured by the MTT method at different concentrations (from 0 to 0.25 mg·mL^−1^). The results confirmed that even if the concentration of the compound reached 0.25 mg·mL^−1^ under non-illuminated conditions, L929 cells and HeLa cells still had a high survival rate and the results indicated that these two compounds had no obvious dark toxicity.

PDT was evaluated by investigating the cytotoxicity of porphyrin derivate at varying concentrations (0 to 0.25 mg·mL^−1^) against L929 cells and HeLa cells under red light irradiation (Figure 6b). The results revealed that THPP and THPP-(PNIPAM-*b*-PMAGA)_4_ have very low phototoxicity to L929 cells. However, for the phototoxicity of HeLa cells, the THPP-(PNIPAM-*b*-PMAGA)_4_ was higher than THPP which may be due to π–π stacking and the poor water solubility of THPP. All the decreasing effects of PDT were in aqueous solution to trigger quenching. Figure 6c shows that the toxicity of paclitaxel-coated THPP-(PNIPAM-*b*-PMAGA)_4_ micelles to HeLa cells under light was higher than that of uncoated micelles, which might be due to the death of HeLa cells caused by the release of paclitaxel.

### 3.7. Cellular Uptake of THPP-(PNIPAM-b-PMAGA)_4_ Micelles

The cellular uptake behavior of THPP-(PNIPAM-*b*-PMAGA)_4_ could be detected by flow cytometry. The cellular internalization of THPP-(PNIPAM-*b*-PMAGA)_4_ micelles in HeLa cells for 4 h and 12 h was shown in Figure 6d. The fluorescence intensity of HeLa cells with THPP-(PNIPAM-*b*-PMAGA)_4_ micelles produced a greater shift compared with untreated cell, this indicated mostly THPP-(PNIPAM-*b*-PMAGA)_4_ micelles had been internalized. The results showed that the fluorescence intensity after 12 h treatment was significantly stronger than that after 4 h treatment. This indicated that HeLa cells had an obvious time-dependent engulfment of THPP-(PNIPAM-*b*-PMAGA)_4_ micelles and could be better engulfed by tumor cells.

### 3.8. Recognition Properties of Star-Shaped THPP-(PNIPAM-b-PMAGA)_4_ Copolymers

**Figure 7:**
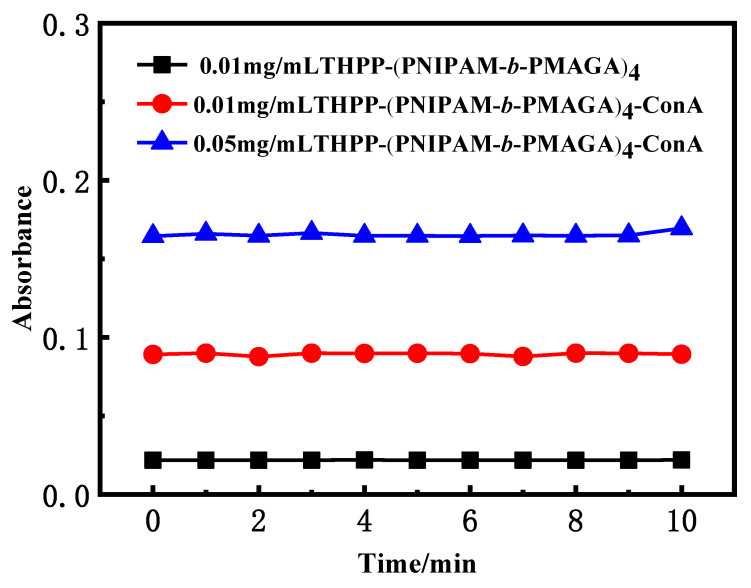
The interactions of Con A (0.5 mg·mL^−1^) with THPP-(PNIPAM-*b*-PMAGA)_4_ at different concentration.

The mutual sugar-protein recognition and specific binding in the living body was of great significance for drug discovery and biomaterial application. Con A could specifically recognize that D-glucopyranoside contains free 3-, 4-, and 6-hydroxyl groups which usually combine with sugar copolymers form Con A cross-linked aggregation. Therefore, the interaction between Con A and THPP-(PNIPAM-*b*-PMAGA)_4_ was studied in an aqueous solution at room temperature. It was found that the turbidity of THPP-(PNIPAM*-b*-PMAGA)_4_ samples increased slightly with the increase in copolymer concentration and there was no precipitation in the solution. It indicated that THPP-(PNIPAM-*b*-PMAGA)_4_ copolymer may combine with Con A to form aggregates. There were no significant changes of Con A aggregation in aqueous which was measured by UV–vis solution turbidity (Figure 7). The specific binding of THPP-(PNIPAM-*b*-PMAGA)_4_ copolymer to ConA in aqueous solution indicated that the sugar copolymer with the core of porphyrin could be used for targeted drug delivery.

### 3.9. Exploration of Paclitaxel Release In Vitro

**Figure 8:**
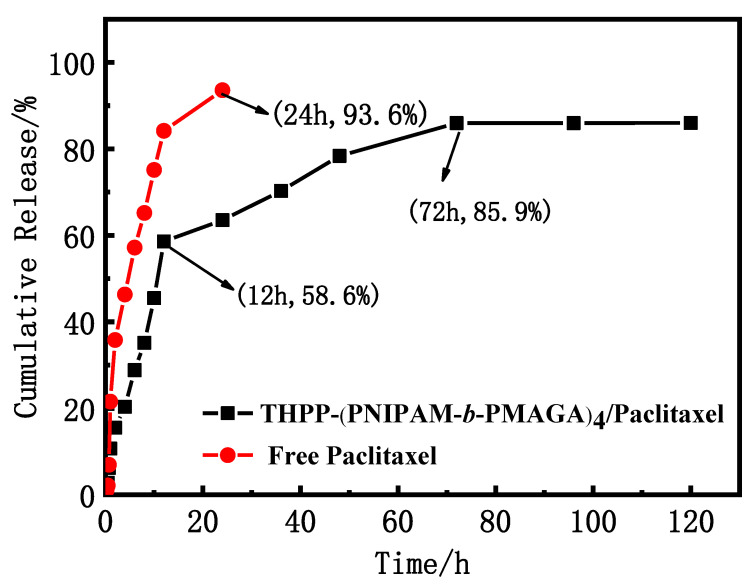
Cumulative release curve of the free paclitaxel and the THPP-(PNIPAM-*b*-PMAGA)_4_/paclitaxel in PBS.

The sustained-release behavior of THPP-(PNIPAM-b-PMAGA)_4_ micelles was evaluated using paclitaxel as the model drug. The encapsulation rate (EE) of the copolymer was about 42.7% by HPLC.The results indicated that the copolymer with a star structure was suitable for drug encapsulation. The two-phase release curves in free paclitaxel and paclitaxel-loaded nanoparticles buffer solutions were observed (Figure 8). It could be found that micelles release of paclitaxel was significantly slower than the free paclitaxel release, followed by a sustained and slower release over a prolonged period of time (72 h, 85.9%). Therefore, the continuous release of paclitaxel-coated nano-micelles could improve its bioavailability and induce cancer cell death to achieve better therapeutic effect.

## 4. Conclusions

The star-shaped double hydrophilic THPP-(PNIPAM-*b*-PMAGA)_4_ with thermo-sensitivity was synthesized by RAFT using porphyrin-cored as a chain transfer agent. The LCST of THPP-(PNIPAM-*b*-PMAGA)_4_ increased with the increasing of the hydrophilic chain segment PMAGA and which could self-assemble into micelles in an aqueous above 37 °C. In vitro experiments of THPP-(PNIPAM-*b*-PMAGA)_4_ showed good biocompatibility and high phototoxicity to HeLa which might be a potential application prospect. In addition, these THPP-(PNIPAM-*b*-PMAGA)_4_ copolymers had a stable Con A aggregation in aqueous solution, so it had a specific ability to recognize Con A. In addition, these block copolymers could be used as nanoscale photosensitizers to further encapsulate hydrophobic paclitaxel.

## Figures and Tables

**Table 1:** Polymerization data of THPP-(PNIPAM)_4_ and THPP-(PNIPAM-*b*-PMA GA)_4_.

Polymer	[M]_0_/[CTA]_0_	Time (h)	*M*_n,GPC_ ^a^	*M*_w_/*M*_n_ ^a^
THPP-(PNIPAM)_4_	50	12	10,500	1.15
THPP-(PNIPAM-*b*-PMAGA)_4_-1	20	12	18,700	1.19
THPP-(PNIPAM-*b*-PMAGA)_4_-2	30	12	22,600	1.21

^a^ Determined by GPC using THF as eluent relative to polystyrene standards. [M]_0_/[CTA]_0_: the molar ratio of monomer to chain transfer agent. *M*_n,GPC_ ^a^: numerical average molecular weight. *M*_w_/*M*_n_ ^a^: molecular weight distribution index.

**Table 2:** Characterization of THPP-(PNIPAM-*b*-PMAGA)_4_.

Polymer	LCST	D_h_ ^a^	CMC ^b^ (mg/mL)	*Φ*_Δ_
THPP-PNIPAM_4_	31.5	——	——	——
THPP-(PNIPAM-*b*-PMAGA)_4_-1	39.9	156.2	0.0005	0.41
THPP-(PNIPAM-*b*-PMAGA)_4_-2	41.9	181.6	0.0025	0.37

^a^ Determined by DLS. ^b^ CMC was determined by a fluorescence spectroscopic method using pyrene as the fluorescent probe.

## Data Availability

The data presented in this study are available in [insert article or supplementary material here].

## Supplementary Materials

The following supporting information could be downloaded at: https://www.mdpi.com/article/10.3390/polym15030509/s1, Figure S1: FTIR spectra of THPP; Figure S2: FTIR spectrum of THPP-(DMP)_4_; Figure S3: ^1^HNMR spectrum of THPP-(DMP)_4_; Figure S4: GPC traces of THPP-(PNIPAM)_4_ and THPP-(PNIPAM-*b*-PMAGA)_4_.

## Abbreviations

NIPAM	N-isopropylacrylamide;
DMP	*S*-1-Dodecyl-*S*′-(α, α′-dimethyl-*α″*-acetic acid) trithiocarbonate;
RAFT	reversible addition-fragmentation chain transfer;
FT-IR	Fourier Transform infrared;
NMR	nuclear magnetism resonance spectroscopy;
GPC	gel permeation chromatography;
DLS	dynamic light scattering.
