# Supplementary material for: A Star-Shaped Copolymer with Tetra-Hydroxy-Phenylporphyrin Core and Four PNIPAM-b-PMAGA Arms for Targeted Photodynamic Therapy
Source: Polymers (Basel). 2023 Jan 18;15(3):509. doi: 10.3390/polym15030509 (PMC9919623; doi:10.3390/polym15030509)
Supplement: Supplementary file 1 [file polymers-15-00509-s001.zip › polymers-2069062-supplementary.pdf]

polymers

Supporting Information

A star-shaped copolymer with porphrin core and four PNIPAM-*b*-PMAGA arms for targeted photodynamic therapy

Changling Liu [1,†], Yirong Wang [2,†], Siyu Wang [2], Pengcheng Xu [2], Renning Liu [2], Dandan Han [2,*] and Yen Wei [3,*]

1 School of Materials science and Engineering, Jilin Institute of Chemical Technology, Jilin City 132022, China
2 College of Biology & Food Engineering, Jilin Institute of Chemical Technology, Jilin City 132022, China
3 Department of Chemistry and the Tsinghua Center for Frontier Polymer Research, Tsinghua University, Beijing, 100084, China
* Correspondence: luckhan2006@163.com (D.H.); weiyen@tsinghua.edu.cn (Y.W.)
† These authors contributed equally to this work.

Preparation and release of paclitaxel-loaded nanoparticles in aqueous solution

The paclitaxel-loaded nanoparticles were prepared by dialysis in aqueous solution. 10 mL distilled water solution of THPP-(PNIPAM-*b*-PMAGA)$_4$ (8 mg) and paclitaxel (2 mg) was gradually added using a microsyringe at 45 ℃ until nanoparticles were formed. They prepared nanoparticle solutions were placed in dialysis bags and dialyzed in 1 L of distilled water to remove the solvent, at 6-hour intervals for 24 hours. Finally, the drug-loaded nanoparticle solution was lyophilized, and the drug loading efficiency of paclitaxel in THPP-(PNIPAM-*b*-PMAGA)$_4$ was determined by high performance liquid chromatography (HPLC). The drug-loaded nanoparticles were immersed in 4ml buffer solution (pH=7.4) and placed in a dialysis membrane bag (MWCO=7000 Da) for dialysis in 26 mL buffer solution. At the set time, 5 mL of drug release solution was extracted and replaced with the same volume of fresh buffer solution and the release of paclitaxel was determined by HPLC.

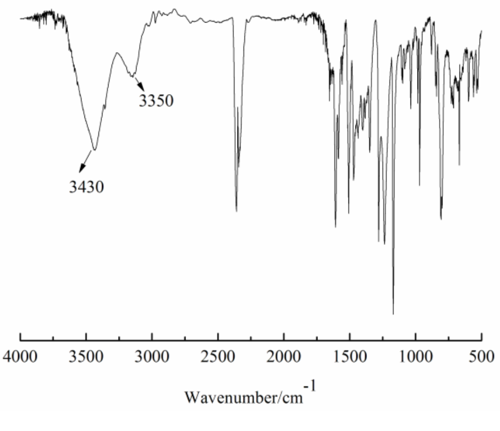

Figure S1. FTIR spectra of THPP.

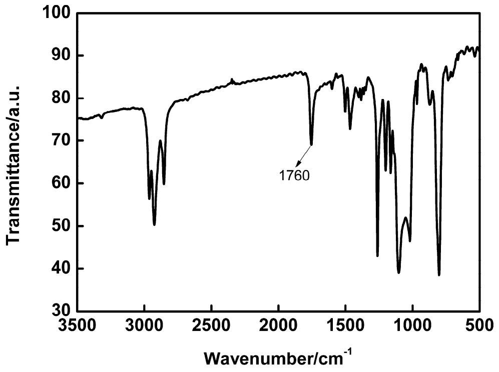

Figure S2. FTIR spectrum of THPP-(DMP)4.

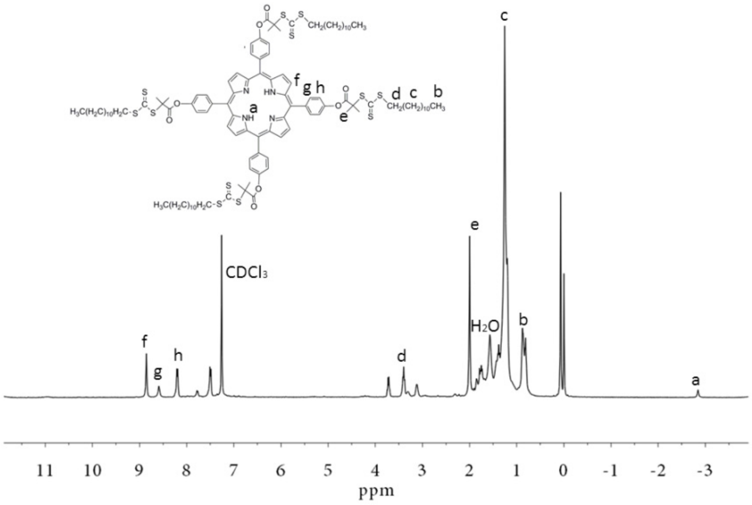

Figure S3. 1HNMR spectrum of THPP-(DMP)4.

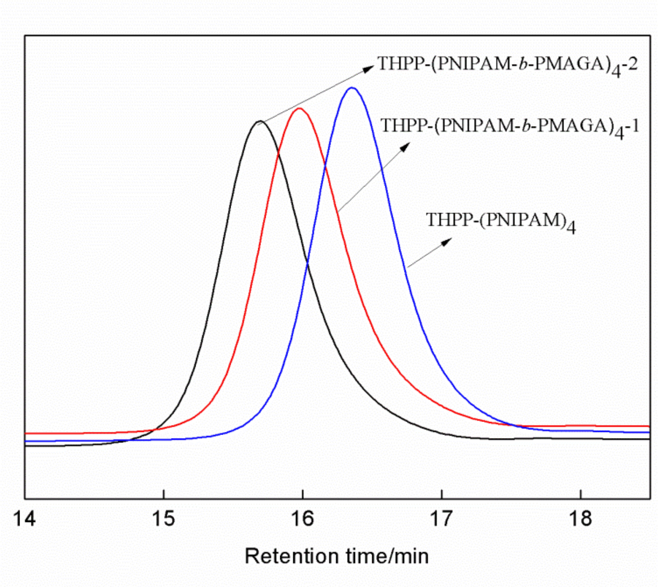

Figure S4. GPC traces of THPP-(PNIPAM)4 and THPP-(PNIPAM-*b*-PMAGA)4.
